# Interference Between Cellular Telephones and Implantable Rhythm Devices: A Review on Recent Papers

**Published:** 2006-10-01

**Authors:** Johnson Francis, Michael Niehaus

**Affiliations:** 1 Department of Cardiology, Calicut Medical College, Calicut, Kerala, India; 2 Department of Cardiology and Angiology, Medical School Hannover, Germany

**Keywords:** pacemaker, defibrillator, implantable, electromagnetic interference, cellular phone, European, American

## Abstract

**Background:**

Cardiac pacemakers and implantable defibrillators are potentially susceptible to electromagnetic interferences as they have complex circuitry for sensing and communication purposes. Cellular telephones being an important source of electromagnetic waves are likely to cause interference in the function of these devices.

**Methods:**

A systematic analysis of studies on interaction between cellular telephones and implantable devices was done using professional databases for literature. Related articles and references of relevant articles were also searched for suitable studies.

**Results:**

Fourteen studies on pacemakers and eight studies on implantable defibrillators were identified. No dangerous malfunction was found in any of the analyzed studies, but most of the studies noted interference with device function when the phone was operated very close to the device. Interference was minimally in those devices with built in feed-through filters for eliminating electromagnetic interference. Device programming and interrogation were the most susceptible phases of operation.

**Summary:**

Cellular phones are likely to interfere with implantable rhythm devices if operated in close proximity or during programming of the device. Patients with implanted devices can safely use cellular phones if they are not carried close to the implanted devices or operated near them. Carrying the cellular phones in the belt position, receiving calls in the ear opposite to the side of the implanted device and keeping the phone as far away as possible while dialing can be considered a safe practice. Interrogation of the devices should take place exclusively in areas where utilization of cellular phones is strictly prohibited. Studies on pacemakers published in the current decade have shown much lesser rates of interference, possibly due to improvement in device technology.

## Introduction

Implantable rhythm device (IRD) is the generic name for the group of implantable devices used for treatment of cardiac arrhythmias like cardiac pacemakers and implantable cardioverter defibrillators. Since these devices have complex microelectronic circuitry and use electromagnetic waves for communication with programmers, they are susceptible to interference from most sources of electromagnetic radiation and magnetic energy [1]. Cellular telephones use radio frequency waves for communication and are likely to interfere with the function of implantable rhythm devices.

Cellular telephones produce both static and dynamic electromagnetic fields. The magnet in the earpiece of the phone produces a low energy static magnetic field. This static magnetic field can activate the internal reed switch causing temporary suspension of sensing function when placed in close proximity to the implanted device [2]. Dynamic fields with much higher intensity are produced by the radio frequency energy used for communication. Today we have two basically different communication systems, analogue and digital systems that vary in their ability to produce interference with IRDs.

## Methods

We carried out a systematic analysis of available data on the interference of implantable rhythm devices by cellular telephones. Database searches were conducted using the search words "cell phone, mobile phone, cellular telephone" in combination with "pacemaker, implantable cardioverter defibrillator, ICD" and independently. The retrieved results were checked to identify relevant studies. Further studies were sought by searching for the related articles and the references of the retrieved articles. Only clinical studies were included.

## Studies Identified

The studies identified are listed in Table 1 [3-24]. The earliest published study dates back to 1995 and the latest was published in 2004. The largest study till date was done by Hayes et al, (1997) with 980 patients with implanted pacemakers [11]. The largest study on patients with implantable defibrillators (ICD) was published in 2002 with 97 patients [19]. A total of 14 studies on patients with implanted pacemakers and 8 studies on patients with ICD were identified [3-24]. Interestingly, majority of the studies were from Europe, with only two from North America and one each from Asia and Australia. There were a total of 3054 patients in all studies taken together.

## Cellular Phones and Networks

Various types of analogue and digital cellular phones are in use across the globe. Analogue telephones transmit by modulation of the amplitude or frequency of electromagnetic waves which are transmitted continuously. On the other hand, the digital telephones transmit data in series of pulses or fast bursts. The advantage of the digital systems is that they allow simultaneous transmission of messages of different users on the same frequency which increases the capacity of the transmission channels. Digital phones are more likely to interfere with IRDs than analogue phones. This is because the pulse repetition rate of the devices falls within the frequency range of physiological signals.

Different frequencies and technologies are in use in different parts of the globe. European system is GSM (Global System for Mobile Communications) in three different frequency ranges. The digital D-net works on a carrier frequency of 900 MHz and the digital E-net works on a carrier frequency of 1800 MHz. The C-net working analogue on 450 MHz was being used in European countries earlier [25]. GSM networks are in use in Asia and Australia [1,7]. The NADC-phones (North American Digital Cellular) work on a carrier frequency of 835 MHz [2].

## Feed Through Filters

Feed through filters are broadband filters using ceramic capacitors which reduce the influence arising from radio frequency sources on pacemakers and ICDs significantly. All IRDs have a titanium can which acts as an electromagnetic shield and a hermetic barrier to protect the internal components from body fluids. The lead wires which carry the pacing pulses and sense cardiac activity may also act as an antenna that conducts undesirable radio frequency signals from cellular phones to sensitive internal electronic circuits. The EMI filter decouples and shields such signals and prevents them from interfering with pacemaker or ICD functions.

## Interference With Pacemakers

Until now, pacemakers constitute the large majority of IRDs and hence most of the studies have been on these devices. Of the 2726 patients included in the various studies, 393 (14.4%) had some form of electromagnetic interference, when the cellular phones were operated in close proximity of the device. But there was considerable heterogeneity between the studies, with the percentage varying from 0 to 43. Inhibition of ventricular output, tracking of the interference sensed in the atrial channel and asynchronous pacing were the common problems noted. Interference could be reduced by programming to lower sensitivity levels [5,15]. Increasing the transmitting power of the cellular phone also increases the probability for interference [5]. In the practical scenario, this occurs in rural areas where access points for the GSM phones are farther apart and the cellular phone automatically steps up the output. The studies uniformly reported no interference when the phone was held in the phoning position over the ear. Almost all the interferences occurred with the phone held directly over the device.

Pacemaker interference by cellular phones has been classified into three groups according to the clinical significance (Hayes et al [11]):

Class I - Clinical responses that are definitely significant. e.g. Interference associated with syncope or pre-syncope
Class II - Clinical responses that are probably significant. e.g. Transient ventricular inhibition less than 3 seconds
Class III - Clinical responses that are probably not significant

In this study, 20% of the total 5533 tests carried out showed interference of some form. Of these 1.7% were Class I, 4.9% Class II and 13.4% Class III interference.

The earliest series in this review was by Barbaro et al [3]. This study involved 101 patients with 43 pacemaker models from 11 manufacturers. 26 patients showed interference at minimum sensing thresholds, with the phone in direct contact with the patient's chest. Pulse inhibition (9.9%), ventricular triggering (19.5%) and asynchronous pacing (7.7%) were the common interferences noted. Maximum distance at which interference occurred was 10 cm with the pacemaker programmed at its minimum sensing threshold.

The study by Hayes et al [11] involving 980 patients with implanted pacemakers was the largest of the lot. It is a well designed study with five types of cellular phones (one analogue and four digital). The telephones were programmed to transmit full power, to mimic the worst case situation. Of a total of 5533 tests conducted, interference was noted in 20%, of which 7.2% were symptomatic. Clinically significant interference was seen in 6.6%. No significant interference was noted when the telephone was placed in the standard phone position over the ear. As expected, interference was much higher when the phone was placed near the pacemaker. Dual-chamber pacemakers were more susceptible (25.3%) than single-chamber pacemakers (6.8%; P<0.001). Pacemakers with feed-through filters were less susceptible to EMI (0.4 to 0.8%) than those without such filters (28.9 to 55.8 %, P=0.01). Marked difference was noted in the incidence of EMI between analogue and digital phones (2.5% vs 23.7%, P=0.01). Interference was higher among pacemaker dependent patients (20.9%) than those who were not (15.2%). The most common types of interferences were tracking interference (14.2%), noise reversion or asynchronous pacing (7.3%) and ventricular inhibition (6.3%). Less common problems noted were atrial inhibition (2.3%), ventricular safety pacing (1.8%), undersensing (0.9%) and rate-adaptive sensor-driven pacing (0.3%). Palpitations was the most common symptom (4.5%). Light headedness occurred in 1.2 % and pre-syncope in 0.2%. Pre-syncope occurred only in those patients who were pacemaker dependent.

Study by Altamura et al [12] included 200 patients. Interference was noted in 21.5 % with GSM phones and 17.5% with Total Access of Communication System (TACS) telephones. Interference was much more common during ringing than on/off phase (131 vs 26 episodes, P<0.0001). Incidence of interference increased with increasing sensitivity (106 at maximum sensitivity vs 51 at basal values; P<0.0001). The authors concluded that if phones were not carried close to the pacemaker, safety was not compromised.

Raden et al [15] reported a study on 144 patients with implanted pacemakers (134 with single chamber and 10 with dual chamber). While 9 patients (6.25%) had intermittent pacemaker inhibition at basal settings, 17 patients (11.8%) showed inhibition on reprogramming to maximum sensitivity. The tests were conducted with the phone directly over the pacemaker site.

Hofgartner et al [8] reported on 104 patients with 58 different models of pacemakers. Interference was noted in 28 different pacemaker types (48.3%) spread over 43 patients (41.3%). Pacemaker inhibition, noise reversion and triggering of pacemaker mediated tachycardia were noted.

**Figure 1:**
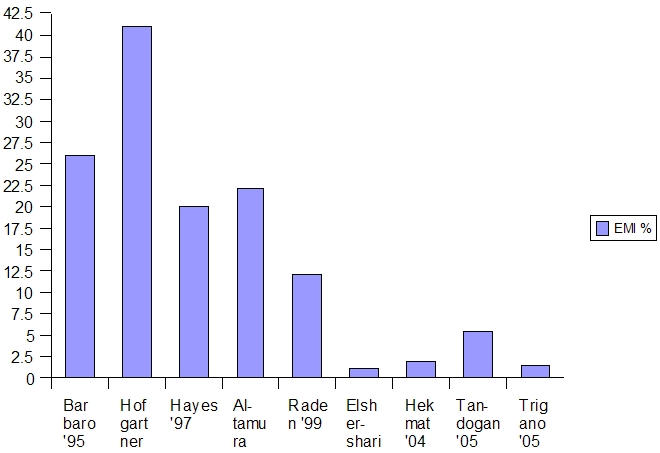
Percentage of EMI in Major Studies on Pacemakers

All the above series with 100 or more patients which reported rather high incidence of interference were from the last decade (Figure 1). The four studies published in the current decade report a very low incidence of interference [21,24]. Smaller studies in the last decade have also reported low incidence [4,6]. In 2002, Elshershari et al [21] reported on 95 patients with pacemakers from 6 different manufacturers. Testing was done with two models of GSM D-net phones. Only one instance of brief oversensing was noted.

Hekmat et al, (2004) [22]  observed pacing inhibition in only 2 patients out of 100, with the phones placed directly above the pacemaker pocket. These inhibitions occurred at programmed sensitivity values of less than 0.5 mV and could be eliminated by reprogramming to 1.0 mV. Hence they recommended programming ventricular sensitivity to 2.0 mV or higher. A change of lead configuration from unipolar to bipolar did not eliminate the interference. All pacemakers in this study were equipped with feed-through filters. All evaluated models showed significant telemetric noise when the phone was placed near the programming head, sometimes even causing loss of telemetric data.

Trigano et al, (2005) [24] noted interference in 1.5% of 330 tests performed in 158 patients. Interference was noted only during 5 tests in 4 unprotected pacemaker models due to interaction with GSM mobile phones. No interference was noted in 12 other tests of identical pulse generator models. The GSM phones had a maximal power output of 2 W and were operating on a 900 MHz carrier frequency.

The largest report of the current decade from Tandogan et al [23] included 679 patients. Interference was noted in 37 patients (5.5%). Thirty-three VVI-R pacemakers were converted to asynchronous mode and 3 were inhibited. One DDD-R pacemaker developed ventricular triggering. Interference was more common when the lead polarity was unipolar (4.12% vs 1.40%, p<0.01). These interferences did not cause any symptoms and the pacemaker function returned to normal when the cell phone was removed away from the patient.

It is likely that better pacemaker technology, especially the use of feed-through filters could have contributed to the lower incidence of interference in the recent studies.

## Interference With Implantable Cardioverter Defibrillators

In the last two decades, ICDs are fast becoming universal arrhythmia management devices for prevention of SCD, especially after the publication of MADIT I and II results. We could identify 8 studies on the influence of cellular phones on ICDs, with a total of 328 patients. 92 patients (28.09%) showed some type of interference when the activated phone was placed over the ICD. Pseudo-oversensing [18], ventricular triggering [17], telemetry noise [16] and partial loss of telemetry [13] were the types of interference noted. No inadequate shock therapy was observed. As in the case of pacemakers, interference occurred mostly when the phone was held close to the implanted device. Devices were most vulnerable for interference during the time of interrogation and programming.

The largest available study was on 97 devices reported by Niehaus et al in 2002 [19]. D-net (900 MHz) and E-net (1800 MHz) phones were used for testing. Interferences (loss of communication or temporary inactivation of the device during interrogation) were noticed in 38 patients. Most of these (93%) occurred while testing close to the device. Jimenez et al [13] published in 1998 their study on 72 patients of which 14 showed interference. Partial loss of telemetry was found in 8 patients with analogue phones and 6 patients with digital phones. But none of these were clinically significant.

Occhetta et al (1999) [16] reported on thirty patients with ICDs from five different manufacturers. Both TACS and GSM phones were used for testing. This study was unique in that it reported interference with all the evaluated models. The interference consisted of noise in telemetric transmission when the phone was located near the ICD and the programmer's head. The noise was most significant during call and reception, leading to loss of telemetry in most cases.

It is important to note that there was no false arrhythmia detections during the tests, neither a delay in recognition of induced ventricular fibrillation. Hence they suggested that patients with implanted ICDs may use cellular phones, but not during ICD programming and interrogation. In contrast to the above report, Fetter et al [14] who studied the effect of North American Digital Communications (NADC)/Time Division Multiple Access-50-Hz (TDMA-50) digital phones on ICDs, reported no interference due to oversensing of the dynamic electromagnetic field in their 41 patients. However, they found that the static magnetic field of the phone's earpiece placed over the ICD will activate the internal reed switch causing temporary suspension of ventricular tachycardia and fibrillation detection.

Chiladakis et al [18] reported on 36 patients with ICDs from two manufacturers. In seven devices from one manufacturer, they noted transient EMI causing 19 erroneous sensing events (pseudo-oversensing) when the phone was operated close to the programmer head. But these events were not logged as arrhythmia episodes by the counter in the device. Therefore this observation has to be interpreted as adverse interaction between the phone and the telemetry function of the ICD. No interference in the function of the ICD was documented regardless of the distance, power or mode of operation of the cellular phone.

## Conclusion

In summary, cellular phones are likely to interfere with implantable rhythm devices if operated in close proximity or during programming of the device. Patients with implanted devices can safely use cellular phones if they are not carried close to the devices or operated near them. Carrying the cellular phones in the belt position, receiving calls in the ear opposite to the side of the implanted device and keeping the phone as far away as possible while dialing can be considered a safe practice. Interrogation of the devices should take place exclusively in areas where utilization of  mobile phones is strictly prohibited as this is the period in which maximum interference is likely. Due to the heterogenic reactions of the implanted devices on cellular phones, EMI by cellular phones should be tested carefully in every new developed implantable rhythm device.

## Figures and Tables

**Table 1:**
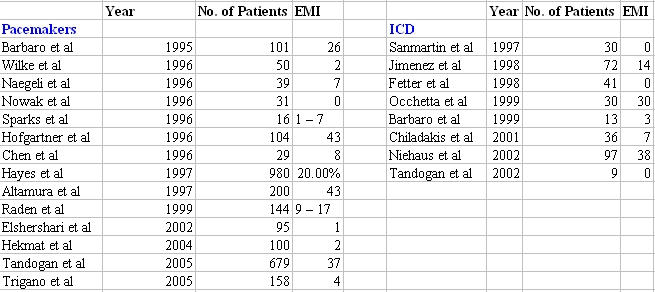
Clinical Studies on Implantable Rhythm Devices and Cellular Phones
